# Thrombus Development on a Transseptal Sheath in the Right Atrium Before Electrical Pulmonary Vein Isolation

**Published:** 2008-02-01

**Authors:** Nieves Romero-Rodriguez, Alonso Pedrote, Eduardo Arana-Rueda, Maria Victoria Mogollon-Jimenez

**Affiliations:** Arrhythmia Unit. Hospital Virgen del Rocio, Seville, Spain

**Keywords:** transseptal catheterization, intracardiac ultrasound, intracardiac thrombus, pulmonary vein, ablation, stroke

## Abstract

We describe the case of a patient who developed a thrombus on the transseptal sheath in the right atrium before transseptal puncture for circumferential pulmonary vein isolation for paroxysmal atrial fibrillation treatment. The use of intracardiac echocardiography allowed to its identification and probably prevented the patient from suffering a serious thromboembolic complication.

## Case presentation

**Figure 1:**
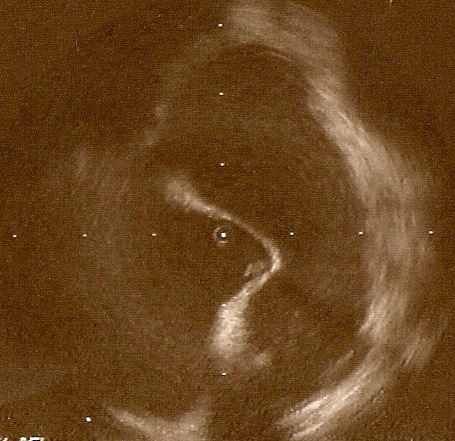
Intracardiac Echocardiography before the jump of the transseptal needle showing "tent formation" in the oval fossa, the transseptal sheath in the right atria and the thrombus (hyperechoic image 10x4 mm) attached to the sheath.

A 47 year-old-man with a paroxysmal atrial fibrillation (AF) and no concomitant cardiovascular risk factors was admitted in our hospital for left atrial ablation. The patient was on warfarin during the previous 8 weeks with INR on admittance of 2.1 together with enoxaparin and the 24 hour-preprocedural transesophageal echocardiography showed no thrombus in the atria. With the use of intracardiac echocardiography (ICE) the oval fossa was localized and an 8 F long sheath was placed nearby. Before being inserted into the left atria with a transseptal puncture, the ICE detected a mobile hyperechoic mass (10x4 mm) attached to the sheath in the right atria and an appearance consistent with thrombus (Figure 1).

**Figure 2:**
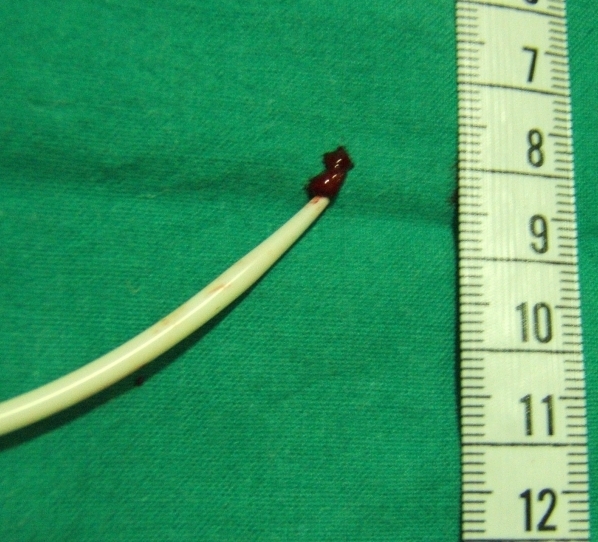
Transseptal sheath containing the thrombus once it had been carefully withdrawn from the right atria.

The procedure was interrupted and the transseptal sheath was carefully withdrawn from the chamber, successfully containing the thrombus (Figure 2) with no overt complications. The procedure was continued 30 minutes later with a new sheath previously immersed in heparin solution and this time the ICE showed no abnormalities. After transseptal catheterization, 5000 I.E. heparin were given intravenously and repeated afterwards in order to maintain activated clotting time (ACT) between 300 and 350 seconds.

The ablation procedure was successfully completed following the circumferential technique. The patient hospital course was otherwise uneventful and after 6 months of follow up no recurrence of AF has been observed.

## Discussion

The demonstration of AF initiation by pulmonary vein depolarization has lead to percutaneous endocardial AF ablation procedures. Currently, the most accepted technique consists in creating circumferential radiofrequency ablation lesions around pulmonary vein ostia with optional additional atrial lesions and with a global arrhythmia control rate reported of >70% of cases in a long term follow-up [1]

Transseptal catheterization provides access to the left-sided cardiac structures from the venous circulation. In this context, ICE has emerged as a useful tool for guiding this catheterization in order to avoid thromboembolic and mechanical complications, even thought its use is not universally established [2].

In fact, thromboembolic events are important complications of these procedures, occurring in 2-4% of patients despite aggressive anticoagulation protocol with heparin. Several series has demonstrated that maintaining an ACT level longer than 300 seconds during the procedure minimized thrombus formation [2].

This case illustrates the ICE usefulness in order to assess the transseptal procedure for prevention of serious complications. In our case, it lead to interruption of the procedure due to the early identification of intracardiac thrombi, and it probably prevented the patient from suffering a systemic thromboembolic complication.
